# 116. Impact of an Antimicrobial Stewardship Program in the First Year of Pandemic 2020 in a Large,Academic, Public Network Hospitals in Bogotá,Colombia

**DOI:** 10.1093/ofid/ofab466.318

**Published:** 2021-12-04

**Authors:** Carlos A Solorzano Ramos, Erika A Ruiz Palma, Gerardo A Muñeton Lopez, Camilo Gomez Rodriguez, Elena V Castro Solarte, Elkin V Lemos Luengas

**Affiliations:** Subred Integrada de Servicios de Salud Sur Occidente E.S.E, Bogotá, Distrito Capital de Bogota, Colombia

## Abstract

**Background:**

Antimicrobial resistance is a major public health threat internationally but, particularly in Colombia. High and increasing rates of carbapenemases are challenging. Implementing antimicrobial stewardship programs (AMSs) in a large, academic, public network hospitals in Bogotá, Colombia.will help curb inappropriate antibiotic use.

Adherence to AMS Program 2020. Subred Integrada de Servicios de Salud Sur Occidente E.S.E Bogotá, Colombia.

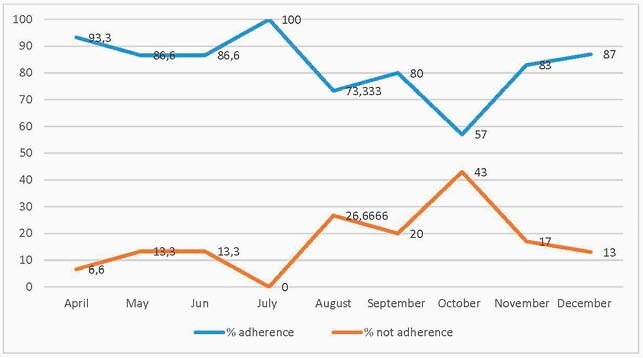

Impact of an Antimicrobial Use Optimization Program in the First Year of Pandemic 2020 in a Large, Academic, Public Network Hospitals in Bogota Colombia

**Methods:**

AMS was established in April 2020 consisting of an administrative champion, Infectious Diseases staff, nurse, General Physician, microbiologist, and pharmacists. Antimicrobial stewardship program interventions included postprescriptive audit and establishment of institutional guidelines. The AMS tracked appropriate drug selection including loading dose, maintenance dose, frequency, route, duration of therapy, de-escalation, and compliance with AMS recommendations. Defined daily dose (DDD) of drugs and health economics evaluations of antimicrobials (April-December 2020). Recommendations are placed in the electronic medical record as a progress note.

**Results:**

From April to December 2020, 1013 patients were evaluated by means of a prospective methodology. Unnecessary 689 days of hospitalization and 4420 days of antibiotic therapy were avoided. Among the top antibiotics discontinued were piperacillin tazobactam for the months of July, August, November and December, while for September and October was meropenem. The intensive care unit was the most frequently intervened service (52%), followed by hospitalization (43%) and the emergency department (5%).Over the course of the year, there was significant adherence to the program, with 100% in July, followed by 93.3% in April, 87% in December, 86.6% in May and June, 83% in November, 80% in September, 73.3% in August and 57% in October. The AMS program was able to save &47.409US in antibiotics and &55.529US in hospitalization, and 11% decrease in nephrotoxicity events (14 renal failures were avoided), which also saved additionally & 23.503 US for a total of an estimated cost saving for the network public hospitals of & 126.441 US by 2020.

**Conclusion:**

Implementation of a multidisciplinary antibiotic stewardship program in this academic, large, academic, public network hospitals in Bogotá, Colombia demonstrated feasibility and economic benefits even in a Covid19 pandemic situation.

**Disclosures:**

**All Authors**: No reported disclosures

